# Oncologic Emergencies

**DOI:** 10.1038/sj.bjc.6601434

**Published:** 2004-01-06

**Authors:** U McGovern, F Bazari, D Chao

**Affiliations:** 1 Royal Free Hospital, UK

The authors have rather undersold themselves by simply calling this a book on ‘Oncological Emergencies’. This book deals with much more and is in fact quite a concise textbook of oncology and certainly a worthwhile addition to any hospital bookshelf.

This is a well-structured textbook that is very easy to read, not just for oncology trainees but all medical and nursing staff. Each chapter starts with excellent introductions to the common problems encountered in oncology patients. Rather than just dealing with the management of emergencies, each chapter covers the physiology and pathology underlying these acute presentations and there are particularly good sections on the neuroanatomy and pathophysiology of pain. With easy-to-follow algorithms and tables, this book provides a very thorough reference in how to manage the symptoms and complications of cancer and its treatment. This would be particularly useful with patients presenting on an acute medical take with no oncology service on site, an increasingly common scenario.

The common emergencies such as neutropenic sepsis are covered in detail, but for the cancer centres and units, guidelines reflecting local practice should already be in place and clearly take precedence. Where these guidelines are not in place, this book would provide an excellent starting point, and of course local guidelines cannot cover every emergency, so this book would make an excellent supplement for those more rare emergencies.

There is an up-to-date section giving background information regarding the different classes of cytotoxic agents commonly used and the side effects that may be encountered. Drugs that may not be familiar to many but can often be extremely toxic are dealt with, and advice is targeted at specific problems they may cause, for example, the management of irinotecan-induced diarrhoea.

Perhaps one of the most useful chapters is the one on acute pain. Often neglected as an ‘emergency’, this is one of the problems likely to cause most distress to oncology patients and in repeated patient surveys this is the one symptom that is consistently poorly managed. Once again, this book sets out very clearly how to start a patient on morphine, how to titrate analgesia and also some insight into the newer opioid-based preparations. This chapter alone should be required reading on every general medical and surgical ward.

We have tried to review this book particularly from the perspective of the SHO and SpR who are more likely to be on the frontline, although with the recent changes in the NHS consultants may find themselves more on the frontline and may find this book equally helpful! It is sufficiently detailed that patients could be managed directly from the pages as it were but remains easy to read. The book covers more than just the emergencies but provides sound advice on the general care of cancer patients and is highly recommended, not just for oncologists, but for all medical, surgical and nursing staff.

